# Knockout of CD8 Delays Reendothelialization and Accelerates Neointima Formation in Injured Arteries of Mouse via TNF-α Inhibiting the Endothelial Cells Migration

**DOI:** 10.1371/journal.pone.0062001

**Published:** 2013-05-02

**Authors:** Jun-Meng Zhang, Ying Wang, Yan-Ju Miao, Yi Zhang, Yi-Na Wu, Li-Xin Jia, Yong-Fen Qi, Jie Du

**Affiliations:** Beijing An Zhen Hospital, Capital Medical University; The Key Laboratory of Remodeling-related Cardiovascular Diseases, Ministry of Education, Institute of Heart Lung and Blood Vessel Diseases, Beijing, China; University of Illinois at Chicago, United States of America

## Abstract

**Objective:**

Delayed or impaired reendothelialization is a major cause of stent thrombosis in the interventional treatment of coronary heart disease. T cells are involved in neointima formation of injured arteries. However, the regulated mechanism of reendothelialization and the role of CD8 T cell in reendothelialization are unclear.

**Methods and Results:**

Immunofluorescence staining showed that CD8 positive cells were increased in wire injured femoral artery of mice. On day 21 after injury, elastin staining showed that knockout of CD8 (CD8^−/−^) significantly increased intimal thickness and a ratio of intima to media by 1.8 folds and 1.9 folds respectively in injured arteries. Evans blue staining showed that knockout of CD8 delayed the reendothelialization area on day 7 after injury (18.8±0.5% versus 42.1±5.6%, *p*<0.05). *In vitro*, a migration assay revealed that CD8^−/−^ T cells co-cultured with WT macrophages significantly inhibited the migration of the endothelial cells (ECs); compared to CD4^+^ T cells, and CD8^+^ T cells could promote the ECs migration. Furthermore, real-time PCR analysis showed that knockout of CD8 increased the level of tumor necrosis factor α (TNF-α) in injured arteries and cytometric bead cytokine array showed that TNF-α was elevated in cultured CD8^−/−^ T cells. Finally, a wound-healing assay showed that recombinant TNF-α significantly inhibited the migration of ECs.

**Conclusion:**

Our study suggested that CD8^+^ T cells could promote the reendothelialization and inhibit the neointima formation after the artery wire injury, and this effect is at least partly dependent on decreasing TNF-α production promoting ECs migration.

## Introduction

Drug-eluting stents (DES) are widely used in the interventional treatment of coronary heart diseases. Although DES use has improved clinical outcome and has dramatically decreased the rates of restenosis than bare-metal stents (BMS)[1] after percutaneous coronary interventions (PCI), the significantly frequent in-stent thrombosis after implanting DES can lead to incidence of fatal myocardial infarction (MI) between 25 and 65% and non-MI-related fatality of 45–75%[2]. The major cause of this treatment failure depends on the intrinsic nature of anti-proliferative, anti-inflammatory and anti-migratory drugs, which impedes vessel healing and leads to incomplete reendothelialization and stent strut coverage [3], [4], [5].

Reendothelialization is critical in modulating local homeostasis, preventing in-stent thrombosis and vessel stenosis after vascular injury [6], [7], [8], [9]. Recent clinical studies have shown the potential value of strategies which targeted to increase endothelial progenitor cells capture and reendothelialization after PCI [10]. Previous studies showed that delayed healing of endothelial cells could lead to increased vascular inflammation, which further contributed to the neointima formation of injured arteries [11], [12], [13]. But whether the inflammatory cells recruited in the injured arteries affect the reendothelialization is not fully understood.

Accumulated evidences indicate that T cells are involved in neointima formation following arterial injury [14], [15], [16], [17], [18]. It was reported that, adoptive transfer of CD8 T cells but not CD4 T cells significantly attenuated the neointima formation in injured arteries of Rag1^−/−^ mice [19]. CD8 expressed on the surface of T cells is essential for the maturation of major histocompatibility complex (MHC) class I-restricted T lymphocytes, which also served as a coreceptor for TCR recognition of MHC class I-associated peptides [20] and mediated cytotoxic T lymphocyte (CTL) activation [21]. However, one report suggested that lack of CD8^+^ T cells does not influence carotid collar-induced neointima formation without denudation of endothelium [22]. Therefore, the objectives of the present study were to investigate the regulated mechanism of reendothelialization and the role of CD8^+^ T cell in reendothelialization in wire injured-arteries of mouse.

In this study, we found that CD8^+^ cells were recruited in the mouse artery 24 hours after wire injury. Knockout of CD8 significantly delayed reendothelialization and increased the injury-induced neointima formation in injured femoral arteries of mouse. Furthermore, our investigation showed that knockout of CD8 inhibited the migration of endothelial cells via increasing secretion of TNF-α *in vitro*. Our results provided evidence for the first time that CD8 could potentially improve the reendothelialization in injured arteries.

## Materials and Methods

### Reagents and Antibodies

The following antibodies were used: anti-CD8 (BD Bioscience, Boston, MA, USA), anti-CD31 (BD Bioscience, Boston, MA, USA), anti-BrdU (Zhongshan Golden Bridge, Beijing, China), macrophage colony-stimulating factor and recombinant human TNF-α were from PeproTech (Rocky Hill, NJ), Evan's blue dye was from Sigma-Aldrich (St. Louis, MO, USA), phycoerythrin-conjugated anti-CD4, FITC-conjugated anti-CD3, isotype antibodies (all from Biolegend, San Diego, CA). Other chemicals and reagents were of analytical grade.

### Animals and Ethics Statement

Male CD8^−/−^ mice and wild-type (WT) littermates on C57BL/6 background were gifted by Jackson Laboratory (Bar Harbor, Maine, USA). All mice used for experiments were within 10 to 12 weeks of age and maintained under specific-pathogen-free conditions in the Laboratory of Animal Experiments at Capital Medical University. The mice were given a standard diet. All animal care and experimental protocols complied with the Animal Management Rule of the Ministry of Health, People's Republic of China (Documentation no. 55, 2001) and the Guide for the Care and Use of Laboratory Animals published by the US National Institutes of Health (NIH Publication no. 85-23, revised 1996) and were approved by the Animal Care and Use Committee of Capital Medical University.

### Wire-Induced Femoral Artery Injury

Wire-induced femoral artery injury was performed as described previously [23], [6] with minor modification. Briefly, male CD8^−/−^ mice and WT littermates, aged 10 to 12 weeks, were anesthetized by use of pentobarbital (50 mg/kg) through intraperitoneal injection. The left femoral artery was exposed by blunted dissection, with femoral vein and nerve carefully separated. The 6-0 silk suture was used to loop the proximally and distal femoral artery in order to stop blood flow temporary. The connective tissues around the artery were removed carefully with microsurgery forceps. The exposed muscular branch artery was dilated by topical application of one drop of 1% lidocaine hydrochloride. Transverse arterioctomy was performed in the muscular branch artery. Then an angioplasty guide wire (0.38 mm) was carefully inserted into the femoral artery from the arterioctomy for more than 5 mm toward the iliac artery. The wire was left in place for 1 min to denude and dilate the artery. After the wire was removed, the proximal portion of the muscular branch artery was ligated by the 8-0 silk suture. Blood flow in the femoral artery was restored by releasing the sutures placed in the proximal and distal femoral portions. The skin incision was closed with a 3-0 silk suture. All animals received 5-bromode-oxyuridine (BrdU) 50 mg/kg intraperitoneally, 18 hrs and 1 hr prior to tissue harvest [24]. For RNA isolation, tissues were frozen in liquid nitrogen.

### Histopathology and Immunohistochemistry

On 1, 3, 7, 14, and 21 day after femoral artery injury, the mice were scarified with intraperitoneal administration of an overdose of pentobarbital (100 mg/kg). Then, the mice were perfused with normal saline followed by 4% paraformaldehyde in PBS (pH 7.4). The femoral artery was carefully excised, fixed and embedded in paraffin. The femoral artery sections (5 µm) were collected at 100 µm intervals (4 sections per artery) placed on polylysine-coated glass slides [6],[25]. For immunofluorescence, frozen arteries sections were incubated with primary antibody (anti-CD8, 1∶200; anti-CD31, 1∶200) and then incubated with FITC-and tetramethylrhodamine isothiocyanate–conjugated secondary antibody (Jackson ImmunoResearch Laboratories, West Grove, PA). Images were captured by the use of a Nikon Eclipse TE2000-S microscope (Nikon, Tokyo, Japan) and analyzed by a person blinded to treatment with use of Image Pro Plus 3.0 (Nikon).

### Elastin Staining

Elastin in the femoral artery was stained with the Gomroris's aldehyde-fuchsin staining method, with elastic fiber stain kit (Maixin. Bio, Beijing, China). In brief, after removing paraffin and rehydrating, sections were incubated for 5 mins in lugol's iodine solution, washed with PBS and then incubated with sodium thiosulfate for 5 mins. After washed with PBS and 70% ethanol, the sections were incubated with aldehyde-fuchsin for 10 mins and acid orange G for 10 seconds.

### Reendothelialization Assay

Reendothelialization of artery was assessed by staining with Evans Blue dye (Sigma) according to the method described previously with minor modification [26]. Briefly, 50 µL of solution containing 5% Evans blue diluted in saline was injected into the tail vein 10 mins before euthanasia, followed by fixation with a perfusion of 4% paraformaldehyde for 10 mins. The ratio between the area stained in blue and the total femoral artery area was calculated.

### Cell Isolation and Culture

Macrophages were isolated from bone marrow of mice and grown in macrophage colony-stimulating factor (M-CSF; Pepro-Tech, Rocky Hill, NJ) as described [27]. Briefly, bone-marrow cells were isolated from femurs and tibias of 8- to 12-week-old mice. Suspensions were cleared of adipose tissue and connective tissue by filtration and then underwent Ficoll-gradient centrifugation to clear residual erythrocytes and non-lymphocytes. Myeloid origin macrophage were cultured in DMEM medium (HyClone, Waltham, MA, USA) supplemented with 10% heat-inactivated FBS in the presence of 50 ng/mL M-CSF. T lymphocytes were isolated from spleen of mice, and then the T Cells from WT mice were purified from the splenocytes using CD8 (Invitrogen, USA) or CD4 cell isolation kit (Miltenyi Biotec, Auburn, CA) according to the manufacturer's instructions. The T cells cultured in complete RPMI 1640 containing 10% fetal bovine serum (FBS) and activated in the presence of anti-CD3 and anti-CD28 (eBioscience, San Diego, CA, USA) for 3 days [25]. Human umbilical vein endothelial cells (HUVECs) were isolated from human umbilical veins as previously described [28] with minor modification. Briefly, HUVECs were digested by type II collagenase (200 µg/100 mL) and isolated by differential attachment rate from other cells and used between passages 3 and 5. The cells were cultured in Endothelial Basal Medium kits (EBM-2; from Lonza, Switzerland) containing 20% fetal bovine serum (FBS, Hyclone). The purity of the HUVECs was more than 98% by calculating the ratio of CD31-positive cell to total nucleus. Human umbilical cords were harvested from Beijing Anzhen Hospital, which was approved by the Ethics Committee of Capital Medical University and gained the informed consent of infants' parents.

### Flow Cytometry Analysis

Deep inguinal region lymph nodes were collected from female WT, CD8^−/−^ mice 3 days after the femoral artery wire injury. Cell suspensions were prepared by standard procedures, subsequently stained with various fluorochrome-conjugated anti-CD4, anti-CD3 for 30 mins, and analyzed by Beckman Coulter Epics XL flow cytometer (Beckman Coulter, Miami, FL) as previously described [22].

### Migration Assay

Migration of ECs was analyzed with wound-healing assay and Boyden chamber methods. For the wound-healing assay, ECs were seeded on 12-well plates and grown up to 90% confluence in complete ECs medium [6]. A proportion of cells was removed from the monolayer with a sterile pipette tip (about 0.5 mm in width), and the wound images were captured at the beginning and at a variety of time points. Images were quantified to compare the migration rate of different cell groups with IPP6.0 image analysis software at 200×field. Cell migration was also quantitated in duplicate by use of 24-well Transwell inserts with polycarbonate filters (8-µm pore size) (Corning Costar, Acon, MA) [27]. ECs in complete media were trypsinized and suspended at (5×10^3^ in 250 µL DMEM high-glucose medium/1% FBS) was added to the upper chamber of the insert. The lower chamber contained WT or CD8^−/−^ activated T cells with or without WT macrophages (1.0×10^5^) in 500 µL RPMI 1640 medium/1% FBS. The plates were incubated at 37 °C in 5% CO_2_ for 12 hrs. Cells that had migrated were counted by use of DAPI staining.

### Cytokine Analysis

To determine the production of TNF-α, monocyte chemoattractant protein-1 (MCP-1), interleukin-6 (IL-6) in macrophages and T cell, WT or CD8^−/−^ activated T cell with or without WT macrophages were plated at 1×10^5^ per well in 24-well plates and cultured for 48 hrs. After incubation, 50 µL supernatant from each sample was incubated with the CBA Flex Set beads assay for 2 hrs [27]. The fluorescence produced by the beads was measured on a FACS Calibur flow cytometer (BD Biosciences) and analyzed by the associated software.

### RNA Extraction and Real-time PCR Analysis

RNA extraction was performed as described [25] with minor modification. RNA was extracted with Trizol reagent (Invitrogen, USA). Aliquots of 2 µg total RNA were used for first-strand cDNA synthesis with moloney murine leukemia virus reverse transcriptase (Promega, Southampton, UK). Aliquots of 2 µL of reaction mixture were amplified with 10 µL SYBR Green PCR Master Mix and 1 µmol/L primers. Table 1 shows the primers used. Amplification was at 95 °C for 5 mins, 95 °C for 45 seconds, and 60 °C for 1 min for each step for 45 cycles. Gene levels were normalized to that of β-tubulin. All samples were run in duplicate.

**Table 1:** Primer sequence for Real time-PCR.

Gene	Forward primer	Reverse primer
TNF-α	5′-GCCACCACGCTCTTCTGTCT-3′	5′-GTCTGGGCCATGGAACTGAT-3′
IL-6	5′-TCCTTCCTACCCCAATTTCC-3′	5′-ACCACAGTGAGGAATGTCCA-3′
MCP-1	5′-CAGGTCCCTGTCATGCTTCT-3′	5′-GTCAGCACAGACCTCTCTCT-3′
IFN-γ	5′-TGCTGATGGGAGGAGATGTCT -3′	5′-TTTCTTTCAGGGACAGCCTGTT-3′
β-tubulin	5′-TCTAACCCGTTGCTATCATGC-3′	5′-GCCATGTTCCAGGCAGTAG-3′

TNF-α, tumor necrosis factor-α; IL-6, interleukin-6; MCP-1, monocyte chemoattractant protein-1; IFN-γ, interferon-γ.

### Statistical Analysis

All values are presented as mean ± standard error of the mean (SEM). The unpaired two-tailed student's t-test was used to assess differences between two groups. Comparison between groups analyzed with one-way ANOVA, then Student-Neuman-Keuls (SNK) multiple comparison analysis by using SPSS Software. A *P*<0.05 was considered statistically significant.

## Results

### CD8^+^ cells were recruited in the injured femoral arteries of mouse

**Figure 1:**
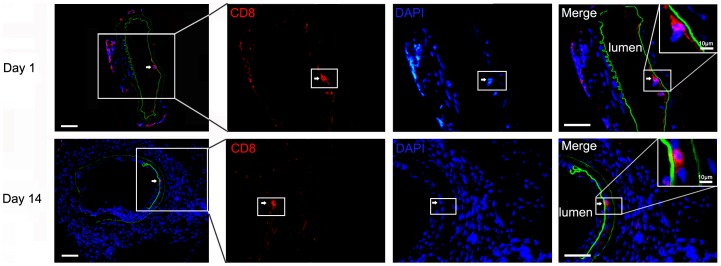
CD8^+^ Cells were recruited in the injured arteries. Immunofluorescence stains for anti-CD8 (red), the elastic lamina (autofluorescence, green) and the DAPI (blue) in the frozen section of 1 day or 14 days injured femoral arteries. Bar = 50 µm.

In the wire-injured femoral arteries of mice, we have performed CD8 immunofluorescence staining and collected images. As shown in Figure 1, CD8^+^ cells were found in arteries on 1 day and 14 days after the injury. The result suggested that CD8^+^ cells were involved in the remodeling of injured arteries.

### Knockout of CD8 increased the neointima formation of injured arteries

**Figure 2:**
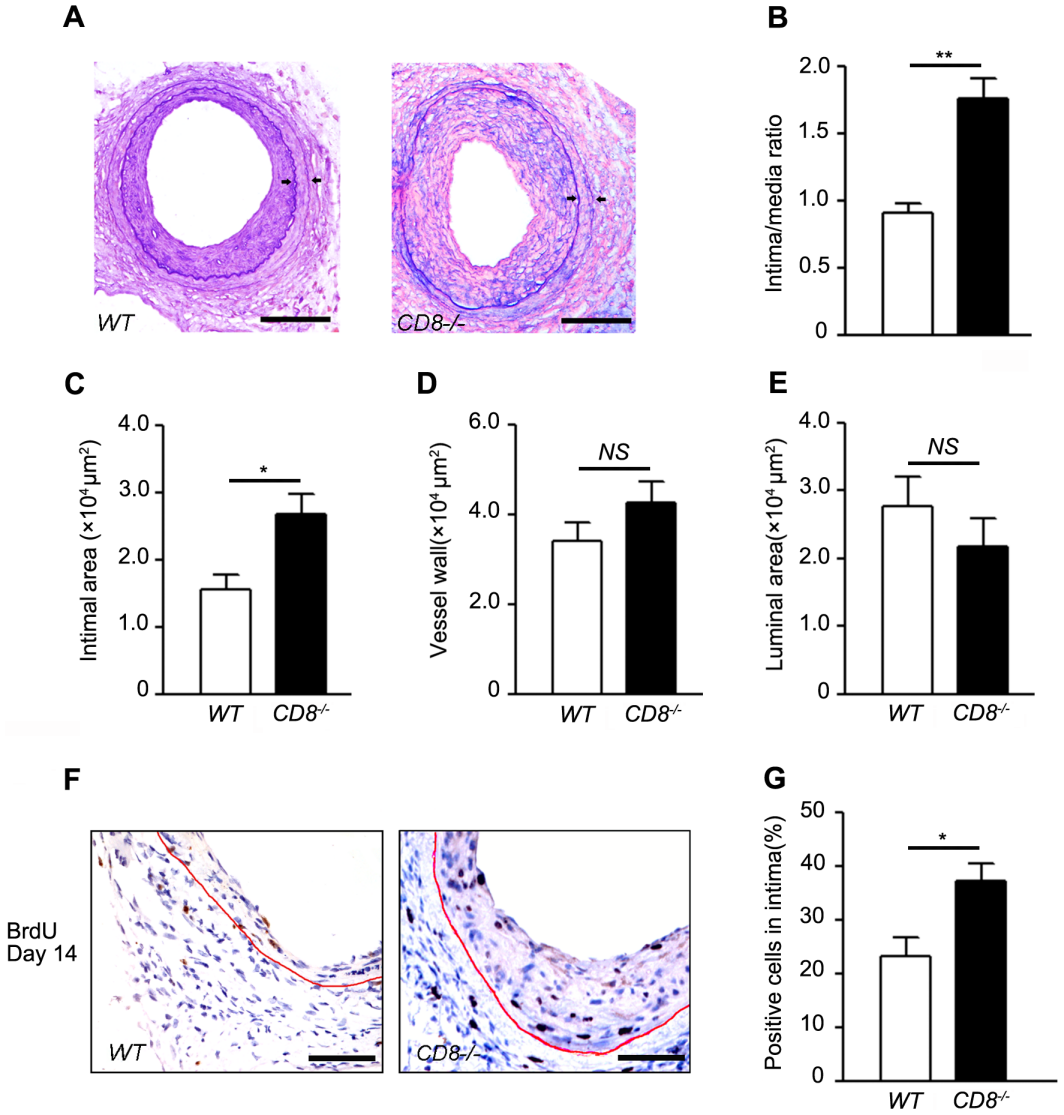
Knockout of CD8 increased neointima hyperplasia in injured arteries. Femoral arteries were injured by a 0.014F guide (0.38 mm) wire and harvested 21 days after surgery for cross-sectioning. A, Representative elastin-stained sections from WT and CD8^−/−^. Bar = 100 µm. B through E, was presented as neointima to media ratio (B), neointima area (C), vessel wall thickness (D), and luminal area (E). Values are mean ± SEM *P<0.05, **P<0.01 versus WT mice, n = 6 each. F, BrdU immunostaining photomicrographs of injured femoral arteries 14 days after injury. G, Quantifications of positive BrdU cells in intima. Bar = 200 µm. Values are mean ± SEM *P<0.05 versus WT mice, n = 6 each.

To explore the role of CD8 in the neointima formation *in vivo*, the femoral arterial wire injury was performed on CD8^−/−^ and WT mice. As shown in Figure 2A, wire injury induced neointima formation at day 21. The elastin staining showed more severe neointima hyperplasia in CD8^−/−^ mice than that in WT mice at day 21 after injury. The ratio of intima to media and intima thickness was increased by 1.9 folds (Figure 2B, *P* <0.01) and 1.8 folds (Figure 2C, *P*<0.05), respectively, in the CD8^−/−^ mice. There were no significant difference in the vessel wall area (Figure 2D) and the luminal area (Figure 2E) between two groups. These results demonstrated the CD8 was attributed to the development of injury-induced neointima hyperplasia.

Then we assessed cellular proliferation by quantifying the incorporation of BrdU in CD8^−/−^ and WT vessels 14 days and 21 days after injury. It was showed that percentage BrdU positive cells accumulation in the intima were increased significantly in CD8^−/−^ mice (Figure 2 F and G, CD8^−/−^ versus WT mice, 37.3±3.2% versus 23.4±3.4%; *P*<0.05, n = 6) at 14 days after injury. However, the percentage BrdU positive cells accumulation in the intima was not significantly different between two groups (Supplement Figure 1, CD8^−/−^ versus WT mice, 17.1±2.1% versus 16.6±2.2%, n = 6) at day 21 after injury.

### Deletion of CD8 delayed the reendothelialization of the injured arteries

**Figure 3:**
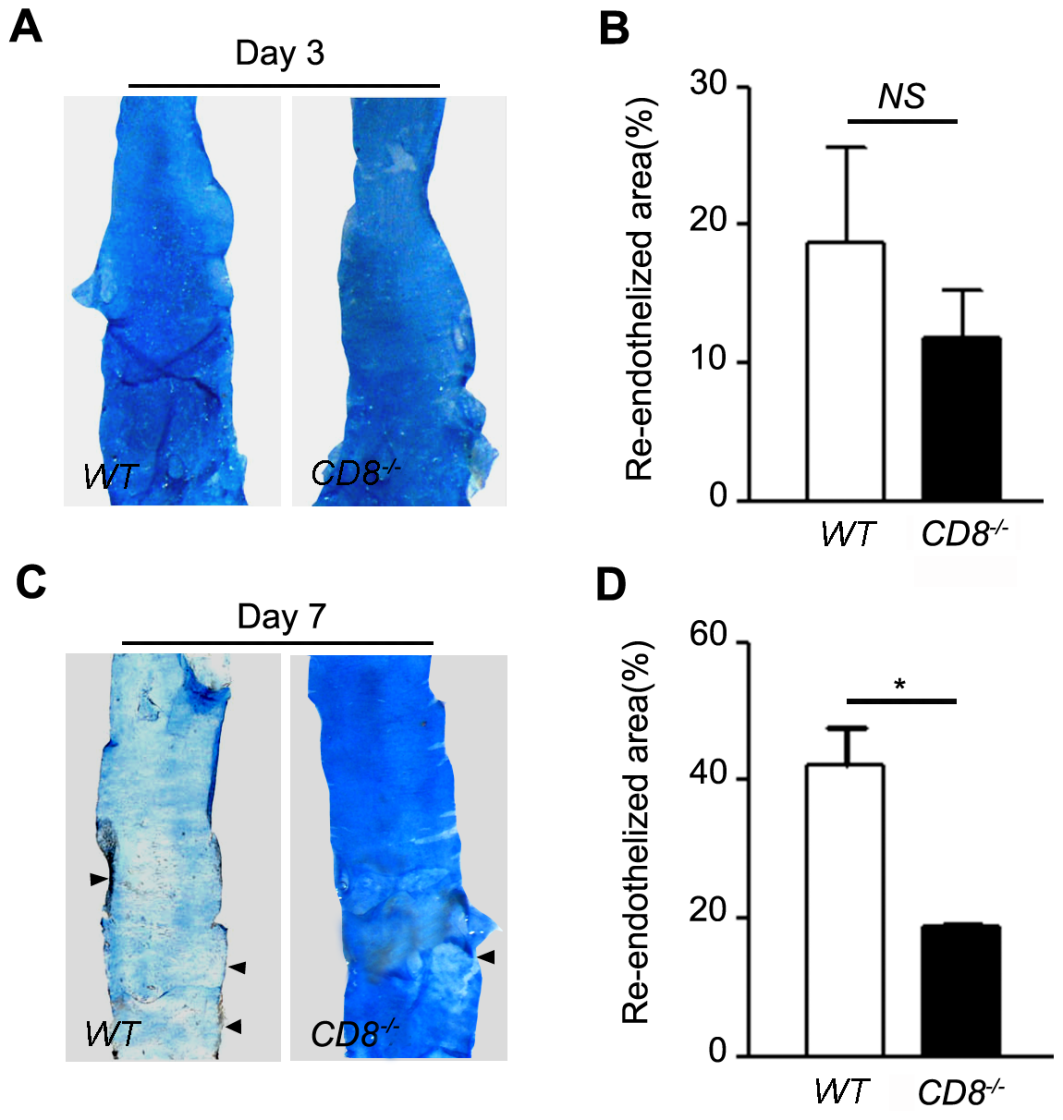
CD8^−/−^ delayed the reendothelialization of the injured arteries. A and C, Evans Blue dye was injected 10 mins before euthanasia on day 3 and 7. The area stained blue corresponds to the area not yet reendothelialized. B and D, Quantification of the reendothelialized areas was performed with computed morphometry (A and C, respectively). *P<0.05 versus WT mice, n = 6 each.

It is well known that rapid reendothelialization after endothelium denudation can inhibit neointima formation [6], [9], at the same time, the proliferation of smooth muscle cells is more quickly in denuded areas than that in endothelial-covered region[9]. To examine the effect of CD8 on the reendothelialization after artery wire injury, Evans Blue dye was used. There was no difference in reendothelialization at the day 3 after injury (Figure 3A, 11.8±3.5% versus 18.8±6.9%, n = 5), however, the reendothelialized area in the CD8^−/−^ arteries was significantly smaller than that in WT arteries (Figure 3B, 18.8±0.5% versus 42.1±5.6%, *P*<0.05, n = 5) at day 7. Moreover, immunostaining with anti-CD31 antibody in transverse sections was used to further demonstrate reendothelialization, at the day 3 point after injury, and only a few CD31^+^ cells were found (Figure S2A); however, more CD31^+^ cells were recruited to the intima in the WT mice than CD8^−/−^ mice at day 7 (Figure S2B), consistent with the result from Evans Blue staining experiment. We found there was no differences in the CD31^+^ length/total lumen length (%) between WT and CD8^−/−^ mice at 21 days after arterial injury (Figure S3, 93.5±3.4% versus 89.2±4.0%, n = 5). Taken together, these results suggested that deletion of CD8 delays the reendothelialization after arterial wire injury.

### Knockout of CD8 inhibited the migration of the endothelial cells

**Figure 4:**
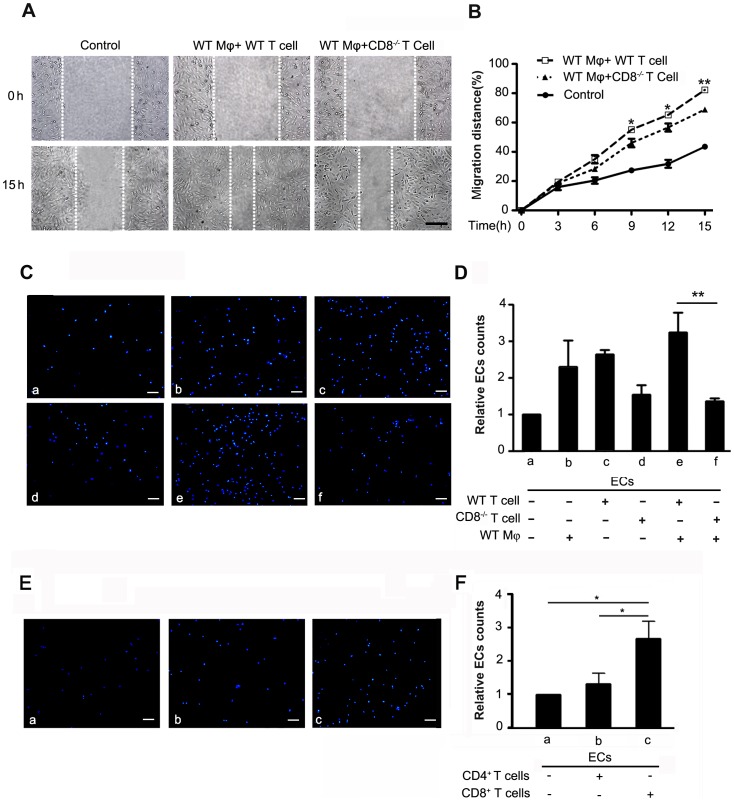
CD8^−/−^ inhibited the migration of endothelia cells. A. Wound-healing assay. The HUVECs monolayers were wounded with a tip and stimulated by the different supernatants (Control, WT Mφ+WT T cell, WT Mφ+CD8^−/−^ T cell). The photos shown were taken at baseline (0 hr) and 15 hrs after wounding, dotted lines indicate the border of ECs. Bar = 200 µm. B. The migration distance of ECs was monitored every 3 hrs for 15 hrs. The data are presented as mean±SEM, *P<0.05, **P<0.01 versus WT Mφ+WT T cell group, n = 6. C, ECs migrated to the lower chamber were counted by DAPI staining. ECs were plated on the upper chamber of transwell inserts. The lower chamber was consisted of co-cultured WT or CD8^−/−^ T cells with WT Mφ. D. Bar graph represents the cell numbers that migrated into the lower chamber. Values are mean ± SEM, **P<0.01 versus WT Mφ+WT T cell group, n = 6,Mφ, Macrophage. E, ECs migrated to the lower chamber were counted by DAPI staining. ECs were plated on the upper chamber of transwell inserts. The lower chamber was consisted of CD4^+^ cells or CD8^+^ cells. F, Bar graph represents the cell numbers that migrated into the lower chamber. Values are mean ± SEM, *P<0.05 versus CD8^+^ cell group, n = 4.

To explore the mechanism that CD8 regulated the reendothelialization, the effect of CD8 on the migration of ECs were first examined. As shown in Figure 4A, the wound-healing assay revealed that the medium from CD8^−/−^ T cells co-cultured with WT macrophages significantly reduced migration of ECs compared to that from WT T cells co-culture with WT macrophages. Figure 4B displayed the time course of percent of migration distance of ECs. The difference between WT T cells co-cultured with WT macrophages group and CD8^−/−^ T cells co-cultured with WT macrophages group was significant at 9, 12, and 18 hrs (1.2±0.7 folds, *P*<0.05; 1.2±0.6 folds, *P*<0.05; 1.2±0.3 folds, *P*<0.01; n = 6, WT T cells co-cultured with WT macrophages group versus CD8^−/−^ T cells co-cultured with WT macrophages group, respectively). In addition, Boyden chamber assay was used to confirm if lack of CD8 inhibits the migration of ECs. Figure 4 C and D, showed that co-culture of CD8^−/−^ T cells with WT macrophages significantly reduced migration of the ECs (4.0±1.2 folds, co-culture of WT T cells with WT macrophages versus co-culture of CD8^−/−^ T cells with WT macrophages, *P*<0.01, n = 6); Although CD8^−/−^ T cells, WT T cells or WT macrophages alone could stimulate the migration of ECs, we did not find significant difference among them.

To investigate whether the CD4^+^ T cells also affect the mobilization of ECs, the Boyden chamber assays were performed using the CD4^+^ T cells and CD8^+^ T cells isolated from the wild type mice. As shown in Figure 4 F and E, CD8^+^ T cells promoted more ECs to traverse the upper chamber than CD4^+^ T cells (2.1±0.1 folds, *P*<0.05, n = 4). Compare to control group, CD4^+^ T cells did not significantly increase the migrated ECs. As a control, T cells were isolated from pooled lymph nodes of injured WT and CD8^−/−^ mice, then stained with antibodies against CD3 and CD4 and analyzed by flow cytometry. There was no difference in the fraction of CD4^+^ T cells in draining lymph nodes from CD8^−/−^ mice at 3 days as compared with that from WT mice (Figure S4, n = 4). Thus, our results indicated that CD8^+^ T cell, but not CD4^+^ T cell, could promote the migration of the ECs. At the same time, interaction between CD8^−/−^ T cells and macrophages was required for inhibiting the migration of ECs.

### Knockout of CD8 promoted the production of TNF-α

**Figure 5:**
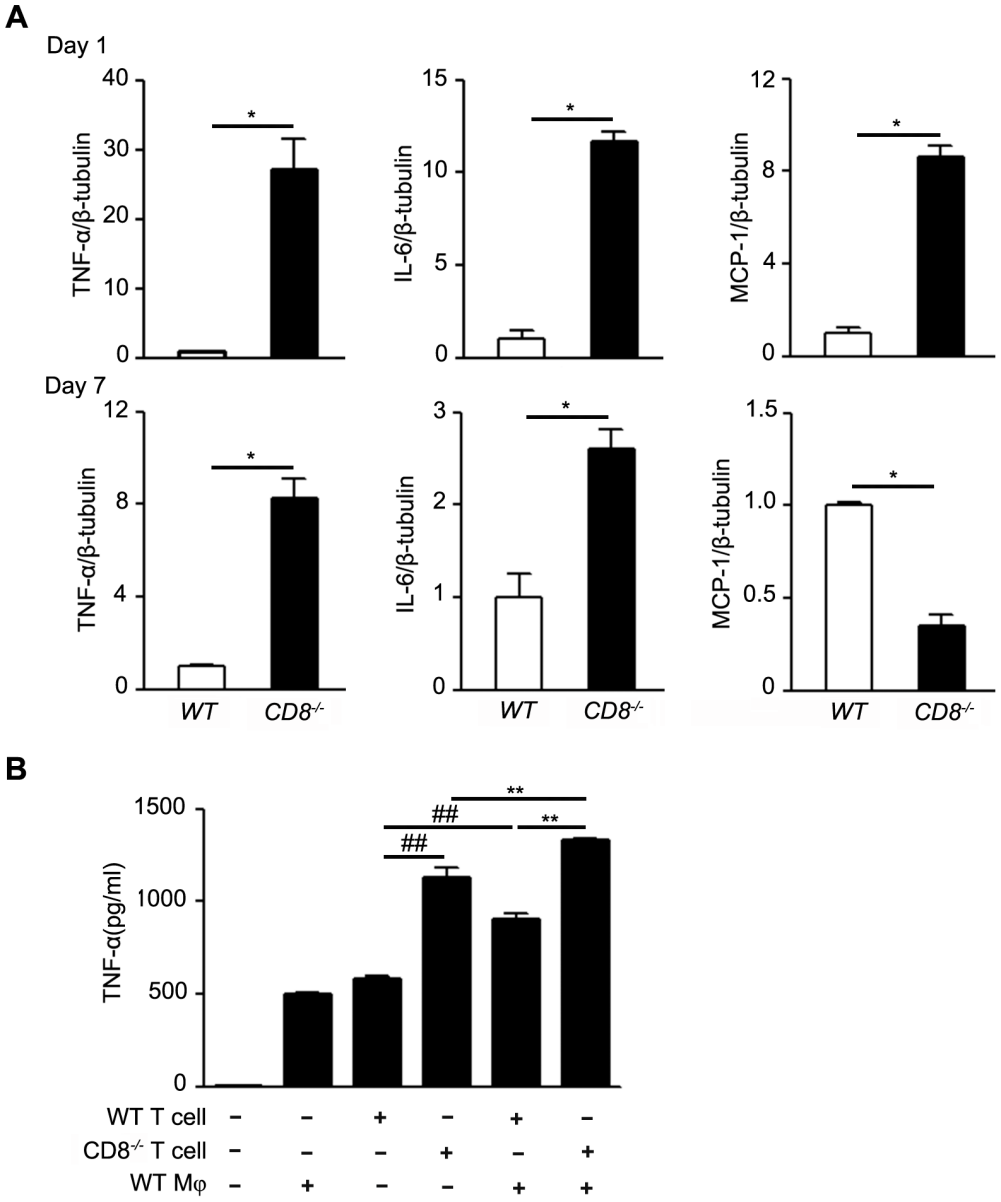
CD8^−/−^ increased the production of pro-inflammatory cytokines. A. *In vivo*, expression of inflammatory factors (TNF-α, IL-6, MCP-1) mRNA in injured femoral arteries from WT and CD8^−/−^ mice at day 1 or day 7 time point after wire injury. Expression of inflammatory factors mRNA was analyzed by quantitative RT-PCR. The level of expression was normalized to that of β-tubulin. *P<0.05 versus WT mice, n = 4. B. *In vitro*, WT T cells or CD8^−/−^ T cells were co-cultured with WT Mφ, and the level of TNF-α production in the medium was measured by CBA. ^##^ *P*<0.01 versus WT T cell group; ^**^ *P*<0.01 versus WT Mφ+CD8^−/−^ T cell group,n = 5. Mφ, Macrophage.

Previous studies showed that inflammatory factors could contribute to the reendothelialization process [11], [12], [13]. To identify the candidate downstream mediators of CD8, the wire injured femoral arteries were harvested, and RT-PCR analysis was performed. Our results delineated that endothelium denudation by wire injury increased the expression of TNF-α, MCP-1 and IL-6 by 27.1 folds (*P*<0.05), 11.7 folds (*P*<0.05), and 8.6 folds (*P*<0.05) respectively, in the CD8^−/−^ femoral arteries compared to that of WT group at 1 day after injury; meanwhile, at the time day 7 after injury, the TNF-α and IL-6 mRNA levels in the CD8^−/−^ mice were still higher than that in WT mice, however the mRNA level of MCP-1 was downregulated in CD8^−/−^ injured arteries (Figure 5A, *P*<0.05, n = 4). Moreover, IFN-γ mRNA level in CD8^−/−^ mice was not upregulated, but downregulated, compared to that of WT mice at the time 1 day after femoral arterial wire injury (Figure S5, 1.0±0.1 versus 0.5±0. 2, *P*<0.05, n = 4).

We found that the co-culture CD8^−/−^ T cells with the WT macrophages could decrease the migration of ECs *in vitro* and the mRNA level of TNF-α was upregulated most significantly in CD8^−/−^ injured artery *in vivo*. It was reported that TNF-α could play a central role in response to femoral wire injury [29], so we next examined the role of CD8^+^ T cells in the production of TNF-α after the co-culture of T cells with the macrophages. We collected the supernatant from the different culture of control (the DMEM with 2%FBS), WT macrophages, WT T cells, CD8^−/−^ T cells, WT T cells with WT macrophages and CD8^−/−^ T cells with WT macrophages, and analyzed by using the CBA cytokine kits. Figure 5B showed that the level of TNF-α produced by CD8^−/−^ T cells co-cultured with WT macrophages was significantly higher than that in WT T cells co-cultured with WT macrophages (*P*<0.01, n = 4). The TNF-α level secreted by CD8^−/−^ T cells alone was higher than that in WT T cells (*P*<0.01, n = 4), however, the co-culture of CD8^−/−^ and macrophages could produce more TNF-α than the T cells alone (CD8^−/−^ T cells co-culture with WT macrophages versus CD8^−/−^ T cells, *P*<0.01, n = 4; WT T cells co-culture with WT macrophages versus WT T cells, *P*<0.01, n = 4).

### Recombinant TNF-α inhibited the migration of the endothelial cells

**Figure 6:**
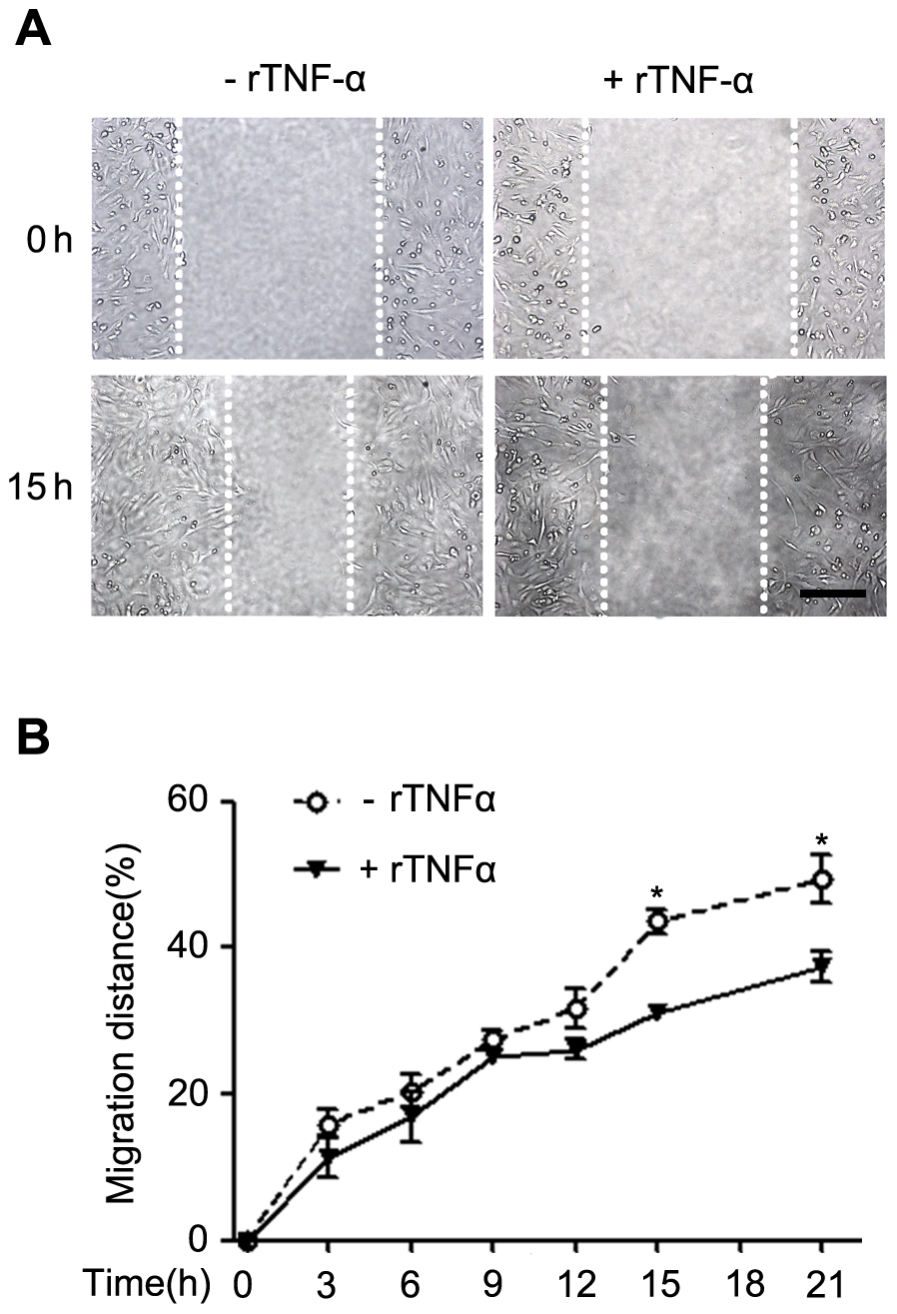
Recombinant TNF-α treatment inhibited the migration of endothelial cells. A. Wound-healing assay. The ECs monolayers were wounded with a tip and stimulated by rTNF-α. The photos shown were taken at baseline (0 hr) and 15 hrs after wounding. Dotted lines indicate the border of ECs. Bar = 200 µm. B. The migration distance of ECs was monitored every 3 hrs for 21 hrs. The data are presented as mean ± SEM. *P<0.05 versus rTNF-α group. n = 4.

To confirm whether CD8 could regulate the migration of ECs through secreting the TNF-α, wound-healing assay was performed *in vitro*. As shown in Figure 6, recombinant TNF-α could significantly depress the migration of ECs at the time 15 hrs (*P*<0.05, n = 4) and 21 hrs (*P*<0.05, n = 4).

## Discussion

Reendothelialization after arterial endothelium denudation plays an important role in preventing in-stent thrombosis and vessel restenosis [6], [9]. In the present study we provided the first evidence that CD8 was essential for the reendothelialization in the injured arteries of mouse. We found that CD8 deficiency could inhibit reendothelialization in response to femoral arterial wire injury, leading to proliferation of SMCs and severe neointima hyperplasia. Mechanically, CD8^+^ T cells, but not CD4^+^ T cells could promote the ECs mobilization. T cells from CD8^−/−^ mice co-cultured with WT macrophages had significantly delayed the migration of ECs *in vitro* with increased secretion of TNF-α.

Arterial stenting is a widely accepted strategy of revascularization which is performed on more than 2 million patients each year. The limitation of bare metal stents is the restenosis that can approach the 40% of the cases in selected patients [30]. It is well known that the damage of endothelium after injury could influence platelet activation and subsequent growth factors secretion [31]. The migration of uninjured ECs precedes proliferation events, and delayed reendothelialization promotes the proliferation and migration of SMCs and intima hyperplasia [6], [9]. Here, impaired reendothelialization was observed in injured arteries of CD8^−/−^ mice, and the SMCs proliferated more quickly than that in WT mice 14 days after femoral wire injury. Meanwhile, the SMCs proliferation showed no difference between CD8^−/−^ and WT mice 21 days post-injury, which may due to the complete reparation of endothelium at that time (Figure S3). Hence, our data suggested that the impaired migration of endothelial cells may be responsible for retarded reendothelialization in CD8^−/−^ mice, and then leads to more severe neointima hyperplasia.

T cell was regarded to play an important role in neointima formation after denudation injury [14], [15], [16], [17], but the role of CD8 in the reendothelialization and neointima formation was unclear. Firstly, the result of flow cytometry excluded the effect that CD8 deficiency may impact on CD4^+^ T cells after the femoral arterial wire injury in mice (Figure S4). Furthermore, in present study we found CD8^+^ T cells could promote reendothelialization and inhibit the neointima formation after arterial wire injury. We also found reduced migration of ECs stimulated by the medium from CD8^−/−^ T cells co-cultured with WT macrophages. Meanwhile there was no significant difference among WT macrophages, CD8^−/−^ T or WT T cells alone on impacting ECs motility. We also performed wound-healing assay and got the similar result that T cells from CD8^−/−^ mouse co-cultured with the WT macrophages could retard the movement of ECs. Therefore, T cells from CD8^−/−^ mouse could inhibit the migration of ECs, which is macrophage dependent.

Recent study showed that transfer of CD8^+^, but not CD4^+^ T cells could reduce neointima formation in Rag-1^−/−^ mice after carotid cuff injury [19]. Saxena reported that lack of CD8^+^ T cells did not influence neointima formation in cuff injured artery [22], while our present study found lack of CD8 promote the neointima formation after arterial wire injury. This discrepancy could be due to the various models, wire injury leading to more severely endothelium damage than cuff injury which primarily induced hemodynamic injury of the vessel.

It is reported that the cytokines play a critical role in the migration of ECs [32], [33]. TNF-α is expressed locally in artery at sites of balloon injury [34], [35] and *in vivo* studies have shown that TNF-α inhibited functional endothelial recovery after angioplasty [11]. In this study, we found that the level of TNF-α was significantly higher in the medium of CD8^−/−^ T cells co-culture with WT macrophages. It is interesting that we also found that the expression of TNF-α was markedly increased in the CD8^−/−^ femoral arteries 1 day and 7 days after injury *in vivo*. Although previous studies have found the upregulation of TNF-α in response to arterial injury[29], [36], [35], this is the first time to report that CD8^−/−^ could increase the production of TNF-α after injury and T cells collaboration with WT macrophages may be one of major resources of TNF-α. TNF-α is a very important cytokine expressed in the injured artery. Previous studies showed that TNF-α contributed to the reendothelialization process [11], but how does TNF-α regulate reendothelialization process remains to be explored. It was reported that TNF-α inhibits functional endothelial recovery after angioplasty [29]. Herein, we performed wound-healing assays and the result showed that rTNF-α could significantly suppress the migration of ECs. This result was consistent with the recent report [32]. Our study also found that CD8^−/−^ could increase the production of TNF-α both *in vivo* and *in vitro*, suggesting that CD8 may regulate the migration of ECs via TNF-α after artery wire injury. In the present study, we also found the expression of IL-6 and MCP-1 in the injured femoral arteries in CD8^−/−^ mice was significantly increased than that in WT mice, but the increases in the mRNA levels of IL-6 and MCP-1 were rapidly reduced to 2.6 and 0.3 fold respectively in injured arteries from CD8^−/−^ mice than that in WT mice at day 7 after injury. It is reported that TNF-α plays a central role in stimulating the expression of IL-6 and MCP-1 in injured arteries [29]. Along with our results, the increases in TNF-α could be essential in regulating inflammatory response in the process of reendothelialization.

Taken together, the present study showed that CD8^+^, but not CD4^+^ T cells could promote the migration of ECs; At the same time, CD8^−/−^ T cells interacting with macrophages could retard the reendothelialization and accelerate the neointima formation, which at least partly depends on increased TNF-α production mediated inhibition of ECs migration. Such findings may reveal the vital functions of CD8^+^ T cells in preventing in-stent thrombosis after PCI.

## Supporting Information

Figure S1 **The proliferation in neointima had no difference between CD8^−/−^ and WT mice at 21 days post-injury.** A, BrdU immunostaining photomicrographs of injured femoral arteries 21 days after injury. B, Quantifications of BrdU positive cells in intima. Bar  = 200 µm, n = 6.
(TIF)

Figure S2 **Re-endothelialization in CD8^−/−^ and WT mice at 3 and 7 days after injury.** A and B, Immunostaining for CD31 in the section of 3-day and 7-day injured femoral arteries of WT and CD8^−/−^ mice respectively. Apparent CD31^+^ positive cell is indicated by arrowheads. Bar = 200 µm.
(TIF)

Figure S3 **Re-endothelialization in CD8^−/−^ and WT mice at 21 days after injury.** A, Immunofluorescence stains for CD31 in the section of 21-day injured femoral arteries. B, The ratio of CD31^+^ length to lumen perimeter in sections were evaluated and averaged on 5 different cross-sections from each artery. Bar = 100 µm, n = 6.
(TIF)

Figure S4 **CD4^+^ T cells in draining lymph nodes after femoral artery wire injury.** T cells were isolated from pooled lymph nodes of injured WT and CD8^−/−^ mice at 3 days after wire injury, stained with antibodies against CD3, CD4 and analyzed by flow cytometry. A, Representative dot plots. B, The percentage of CD4^+^ T cells. n = 4. LN: lymph node.
(TIF)

Figure S5 **CD8 knockout decreased the expression of IFN-γ.** A. *In vivo*, expression of IFN-γ mRNA in injured femoral arteries from WT and CD8^−/−^ mice at 1 day after wire injury. IFN-γ mRNA was analyzed by quantitative real-time PCR. The level of expression was normalized to that of β-tubulin. *P<0.05 versus WT mice, n = 4.
(TIF)
