# Supplementary figures and images for: Knockout of CD8 Delays Reendothelialization and Accelerates Neointima Formation in Injured Arteries of Mouse via TNF-α Inhibiting the Endothelial Cells Migration
Source: PLoS One. 2013 May 2;8(5):e62001. doi: 10.1371/journal.pone.0062001 (PMC3642119; doi:10.1371/journal.pone.0062001)

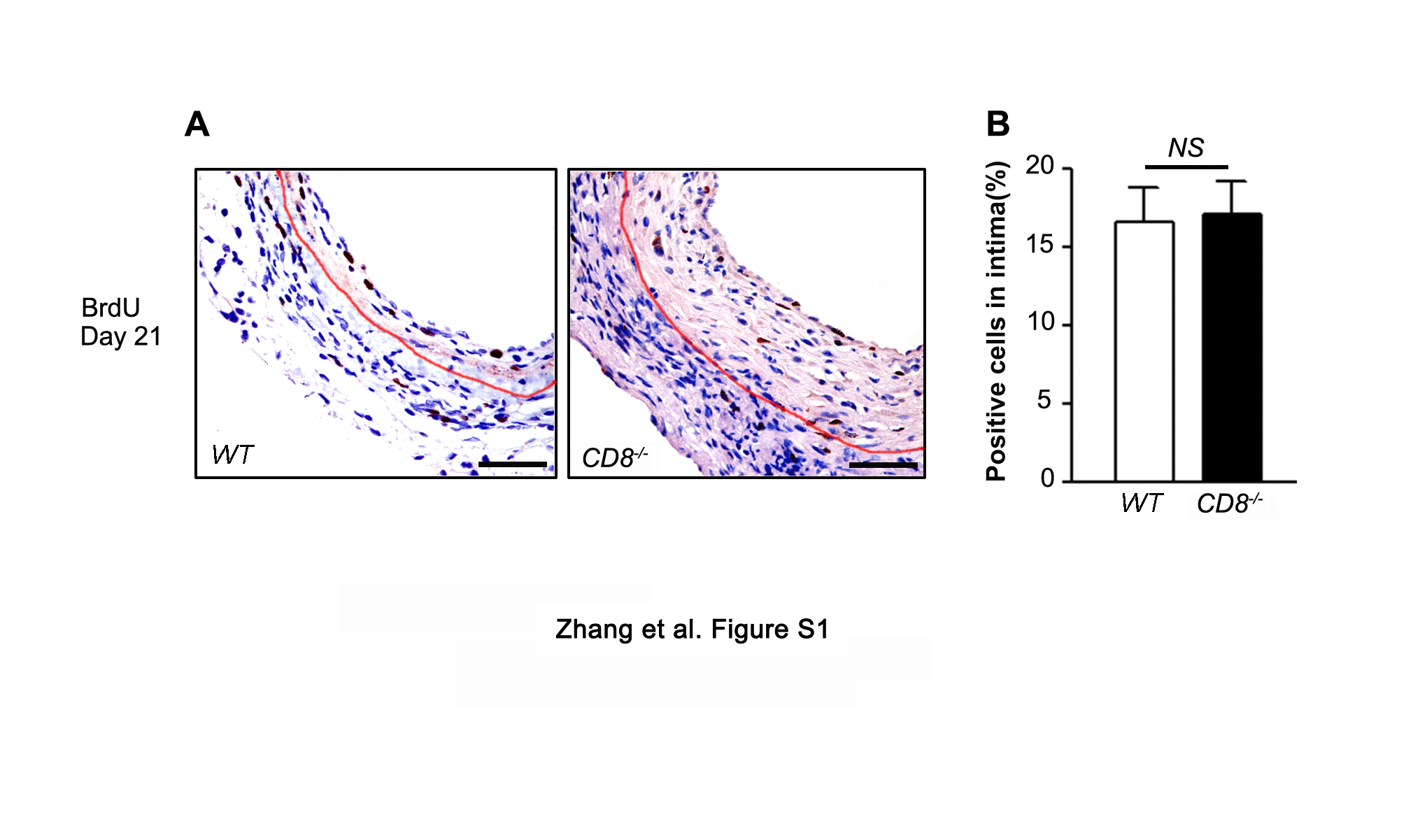

Supplement: Figure S1 — The proliferation in neointima had no difference between CD8−/− and WT mice at 21 days post-injury. A, BrdU immunostaining photomicrographs of injured femoral arteries 21 days after injury. B, Quantifications of BrdU positive cells in intima. Bar = 200 µm, n = 6. (TIF) [file pone.0062001.s001.tif]

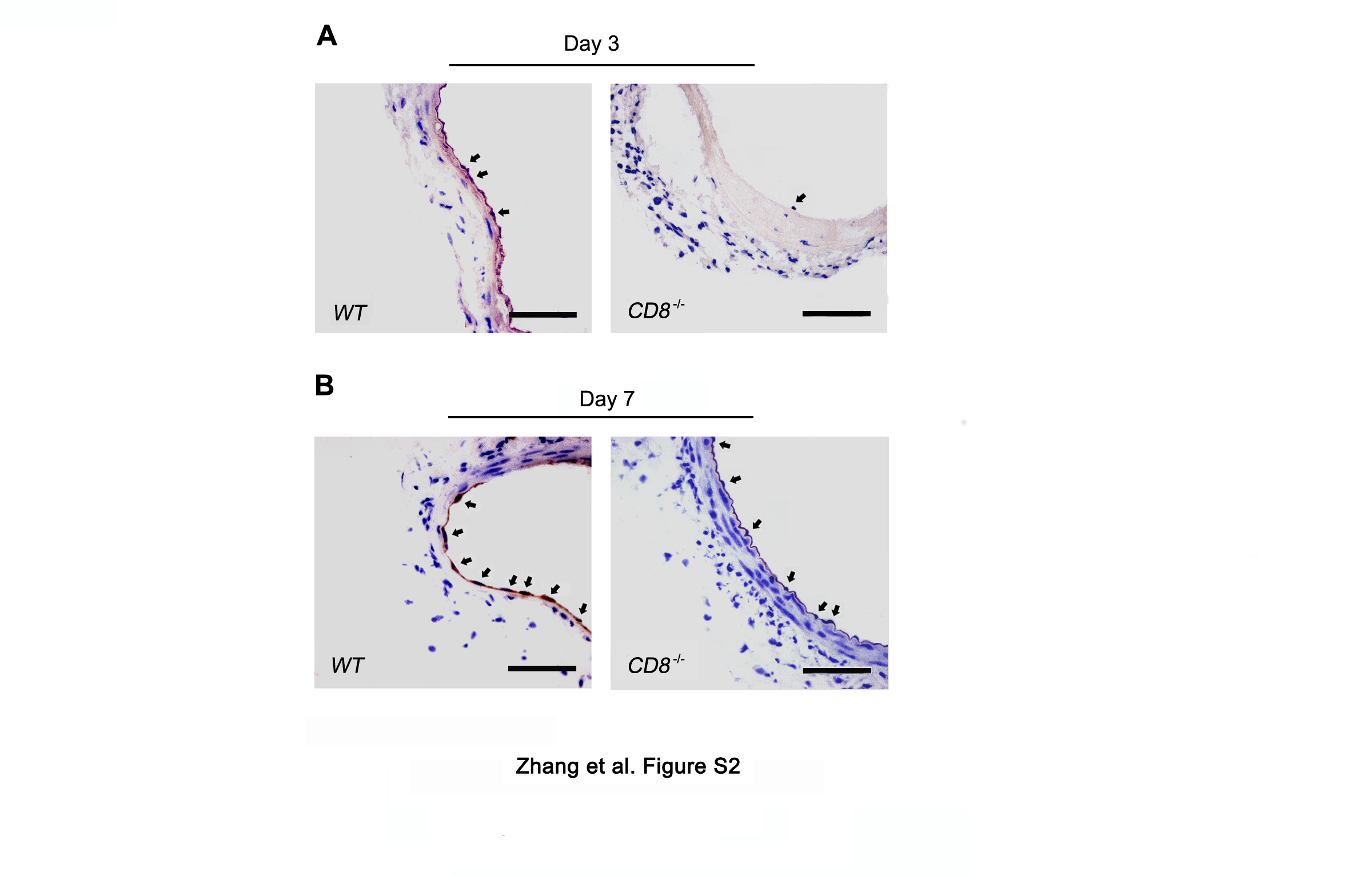

Supplement: Figure S2 — Re-endothelialization in CD8−/− and WT mice at 3 and 7 days after injury. A and B, Immunostaining for CD31 in the section of 3-day and 7-day injured femoral arteries of WT and CD8−/− mice respectively. Apparent CD31+ positive cell is indicated by arrowheads. Bar = 200 µm. (TIF) [file pone.0062001.s002.tif]

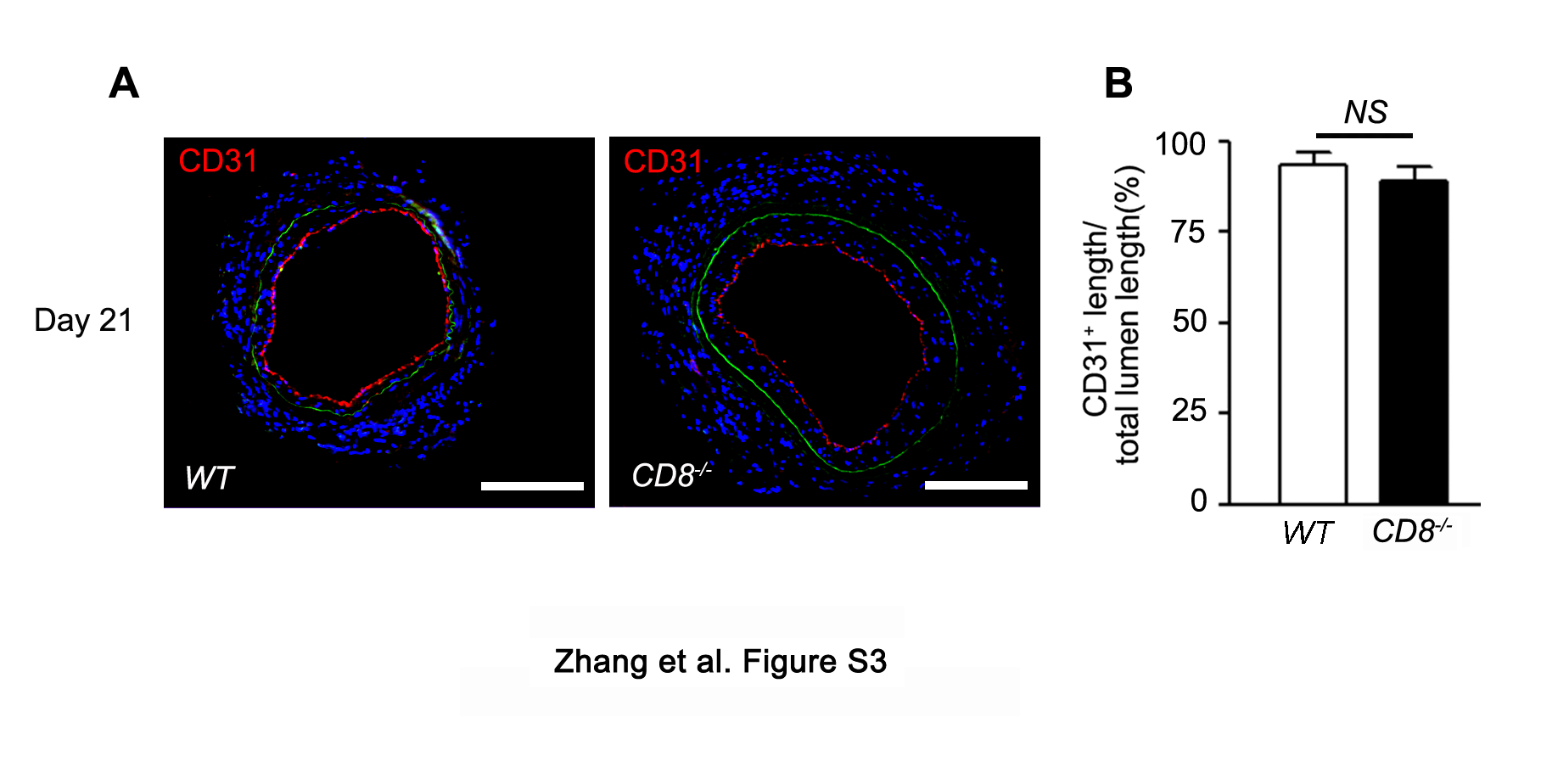

Supplement: Figure S3 — Re-endothelialization in CD8−/− and WT mice at 21 days after injury. A, Immunofluorescence stains for CD31 in the section of 21-day injured femoral arteries. B, The ratio of CD31+ length to lumen perimeter in sections were evaluated and averaged on 5 different cross-sections from each artery. Bar = 100 µm, n = 6. (TIF) [file pone.0062001.s003.tif]

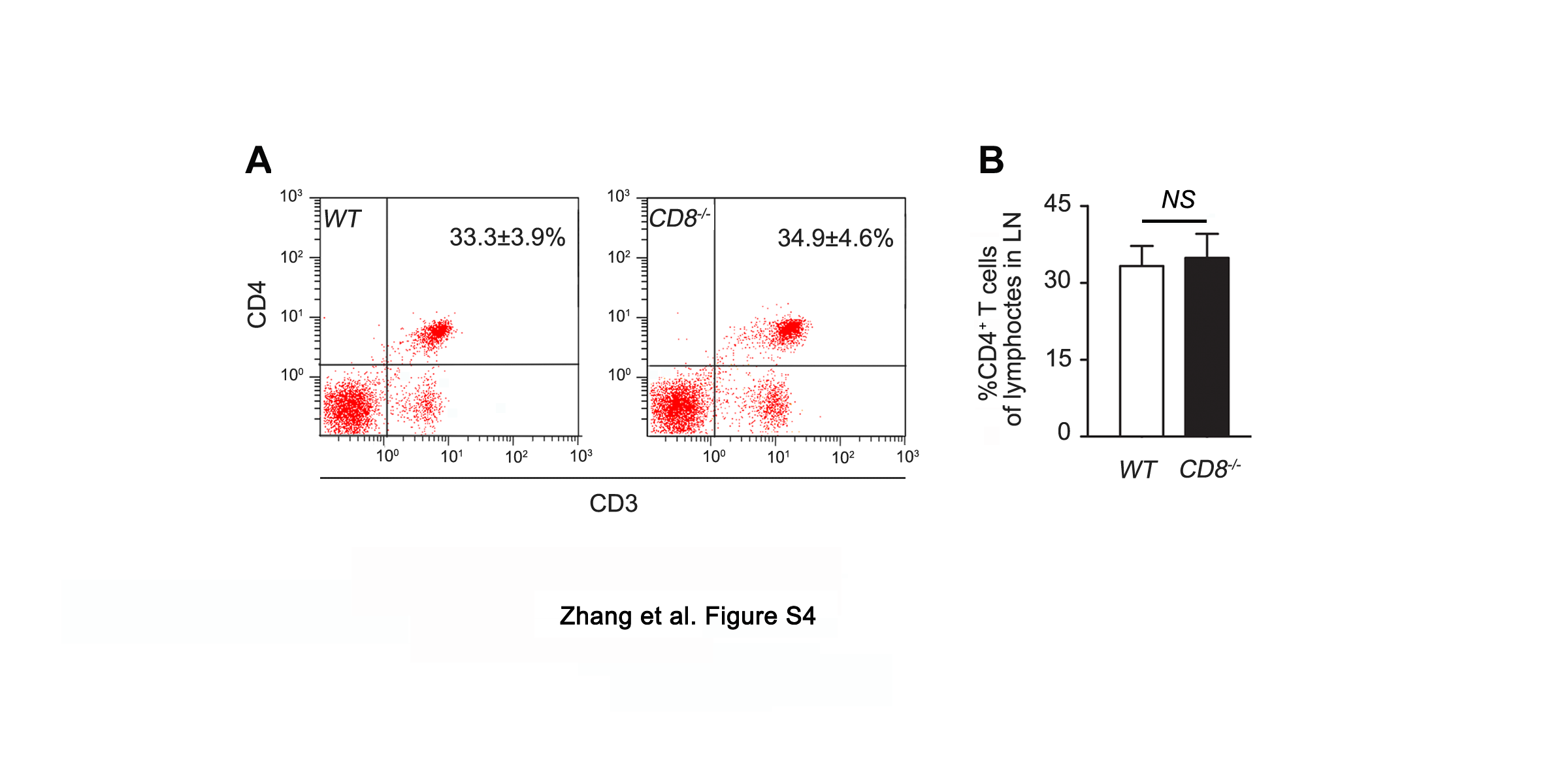

Supplement: Figure S4 — CD4+ T cells in draining lymph nodes after femoral artery wire injury. T cells were isolated from pooled lymph nodes of injured WT and CD8−/− mice at 3 days after wire injury, stained with antibodies against CD3, CD4 and analyzed by flow cytometry. A, Representative dot plots. B, The percentage of CD4+ T cells. n = 4. LN: lymph node. (TIF) [file pone.0062001.s004.tif]

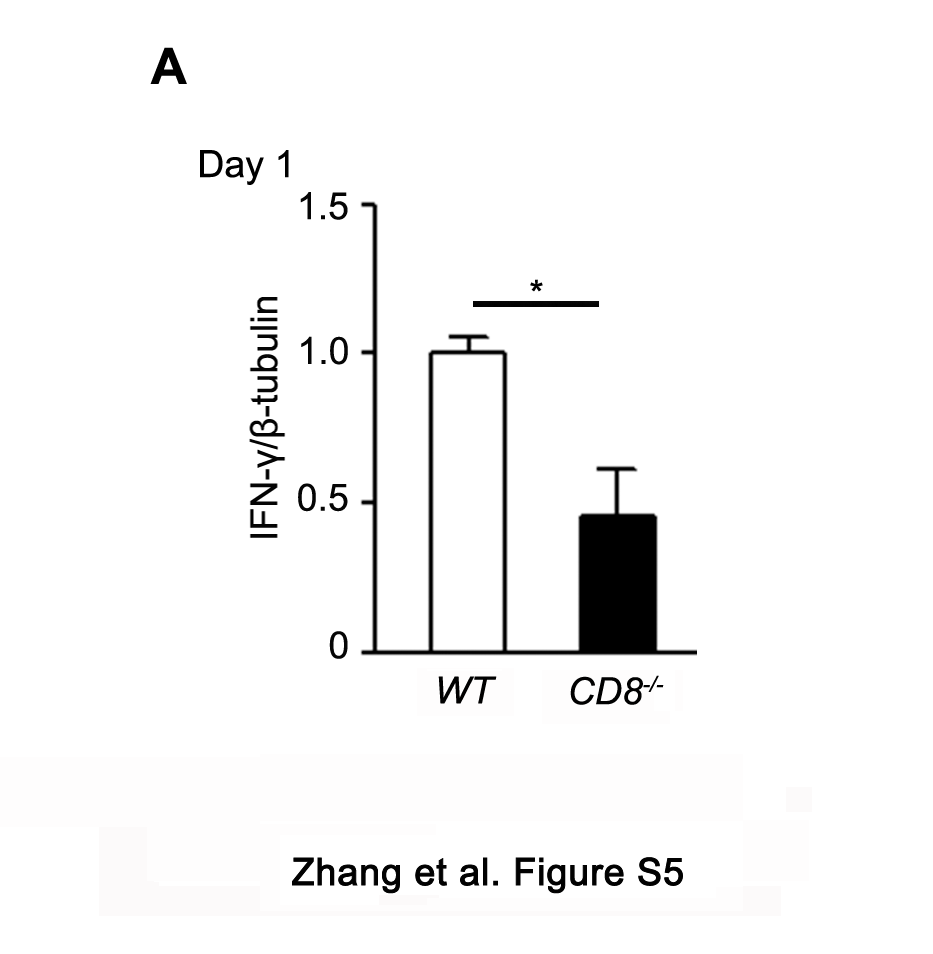

Supplement: Figure S5 — CD8 knockout decreased the expression of IFN-γ. A. In vivo, expression of IFN-γ mRNA in injured femoral arteries from WT and CD8−/− mice at 1 day after wire injury. IFN-γ mRNA was analyzed by quantitative real-time PCR. The level of expression was normalized to that of β-tubulin. *P<0.05 versus WT mice, n = 4. (TIF) [file pone.0062001.s005.tif]
